# Supplementary figures and images for: Blood vessel tortuosity selects against evolution of aggressive tumor cells in confined tissue environments: A modeling approach
Source: PLoS Comput Biol. 2017 Jul 17;13(7):e1005635. doi: 10.1371/journal.pcbi.1005635 (PMC5536454; doi:10.1371/journal.pcbi.1005635)

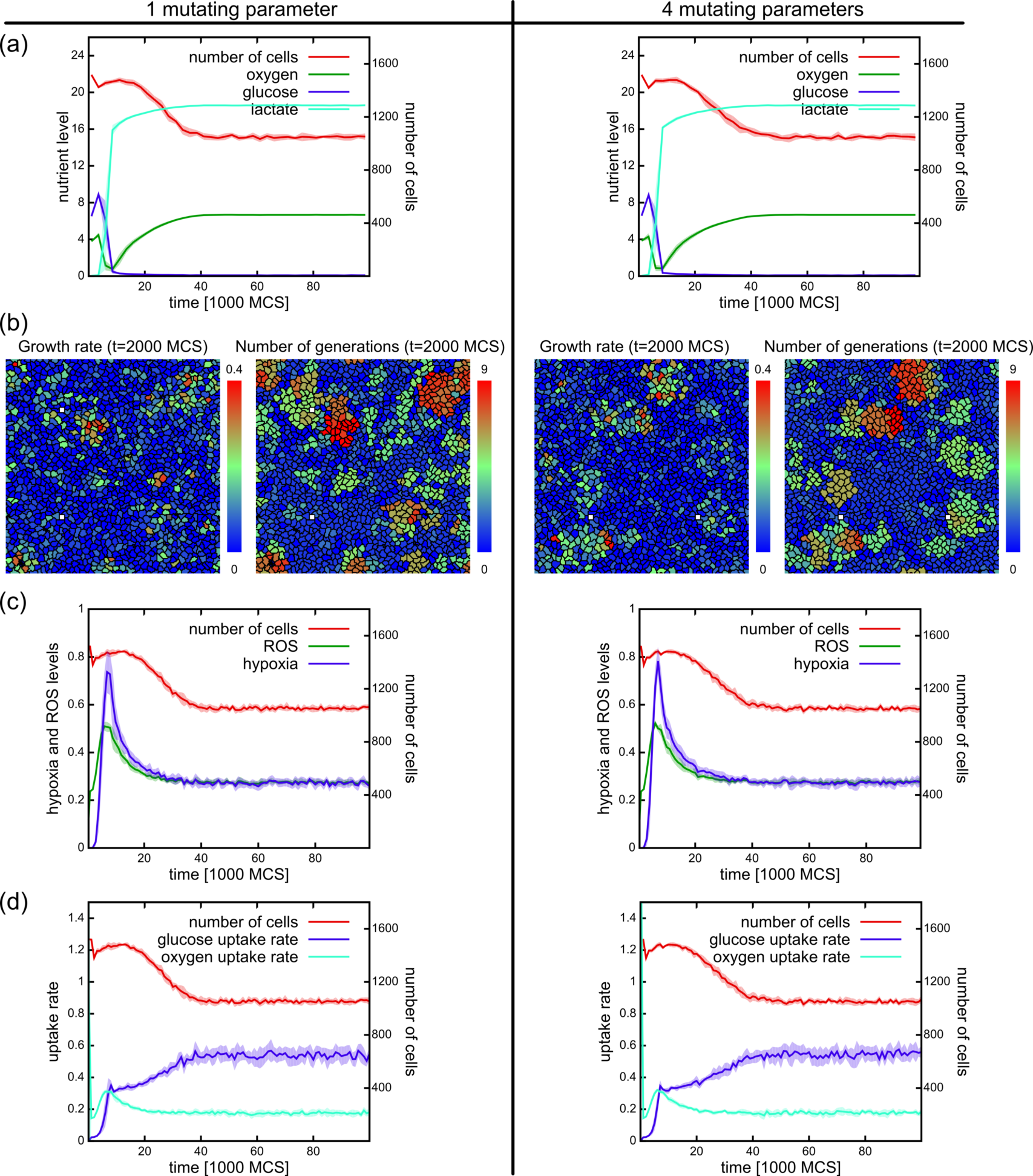

Supplement: S1 Fig — (a) Nutrient levels and number of cells in the simplified models; compare with Fig 3d. (b) Patches of high growth similar to those in the full model shown by the instantaneous growth rate and generation number of cells as in Fig 4e and 4f. (c) Hypoxia and ROS in the simplified models; compare with Fig 4l. (d) Oxygen and glucose consumption in the simplified models; compare with Fig 4m. Population averages from 10 independent simulation runs with standard deviation across simulations. (TIF) [file pcbi.1005635.s001.tif]

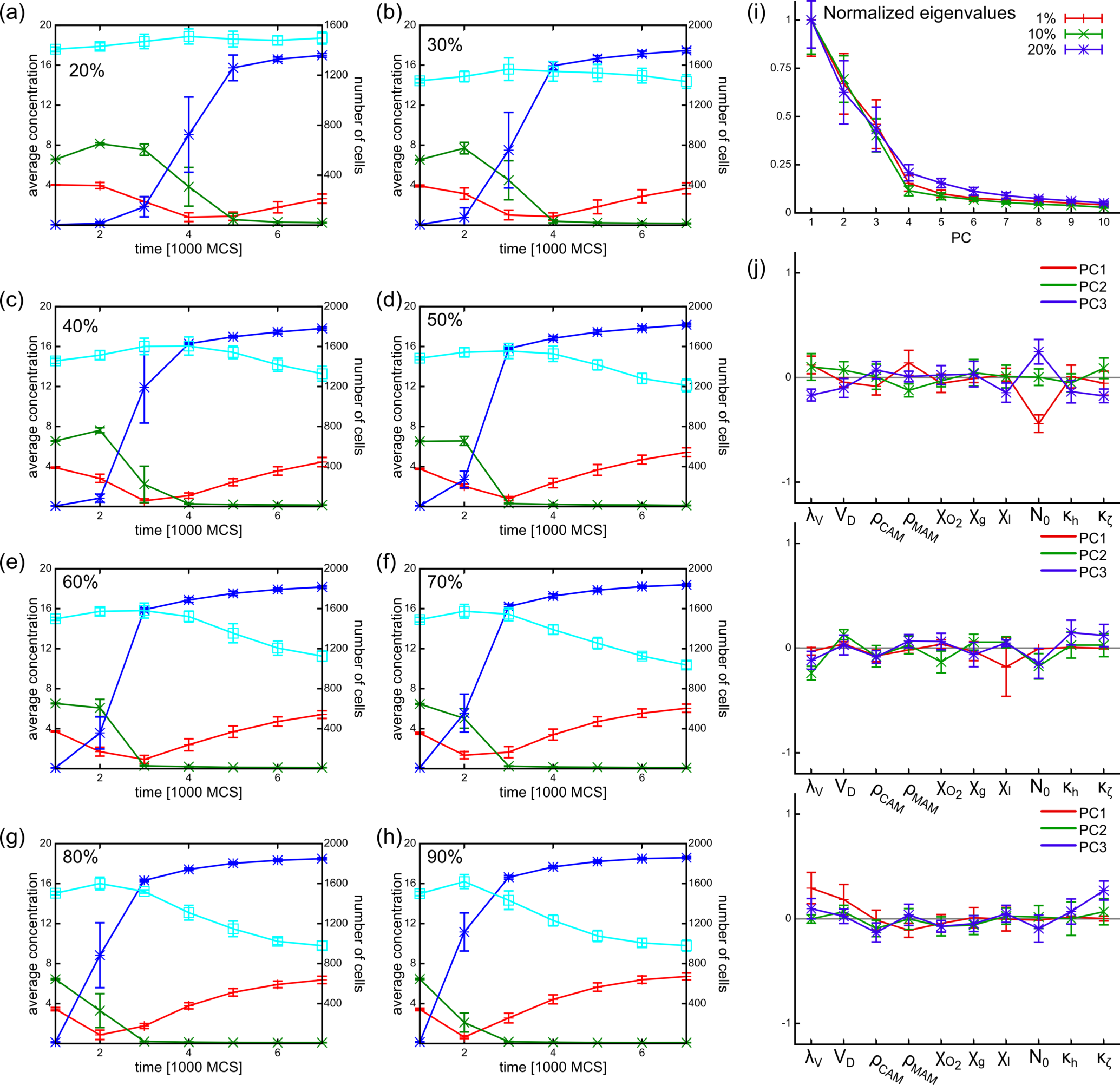

Supplement: S2 Fig — Average number of cells and nutrient concentrations in simulations with mutation rates of 20% (a), 30% (b), 40% (c), 50% (d), 60% (e), 70% (f), 80% (g), and 90% (h). Progress of populations through stages is increasing with increasing mutation rates. Population averages from 10 independent simulation runs with standard deviation across simulations. (i) Relative weight (eigenvalues) of the principal axes of populations in phenotype space averaged from 10 independent simulation runs for 1%, 10%, and 20% mutation rates each. (j) Averaged composition of the first three principal axes in the populations shown in (i). (TIF) [file pcbi.1005635.s002.tif]

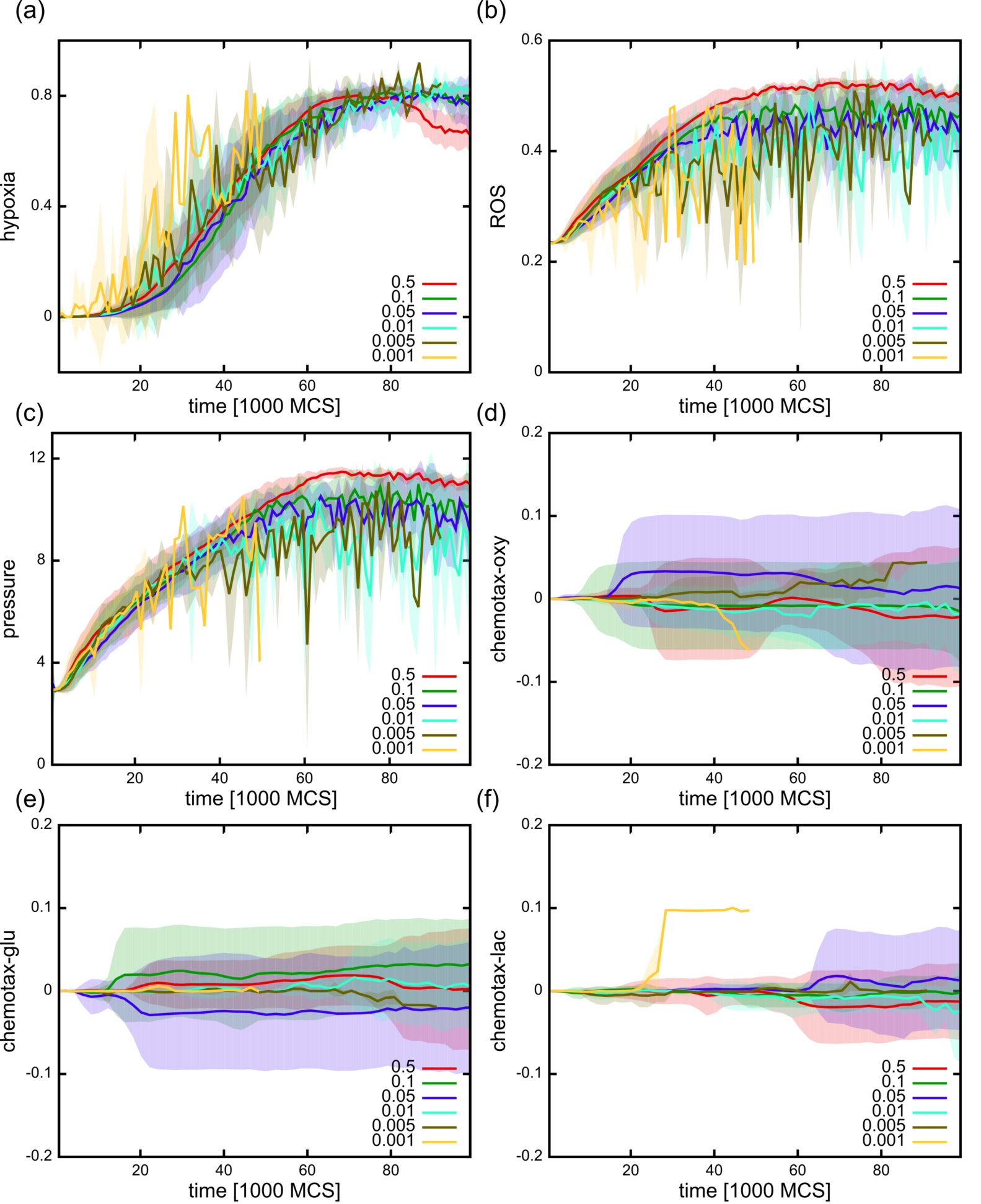

Supplement: S3 Fig — Hypoxia (a), ROS (b), cellular pressure (c), and chemotaxis towards oxygen (d) glucose (e) and lactate (f), remain largely unaffected by different vessel blocking probabilities. Note that the variation increases with lower blocking probability P due to fewer surviving cell populations. Population averages from 10 independent simulation runs with standard deviation across simulations. Blocking probability values P show on graphs. (TIF) [file pcbi.1005635.s003.tif]
