# Supplementary material for: Blood vessel tortuosity selects against evolution of aggressive tumor cells in confined tissue environments: A modeling approach
Source: PLoS Comput Biol. 2017 Jul 17;13(7):e1005635. doi: 10.1371/journal.pcbi.1005635 (PMC5536454; doi:10.1371/journal.pcbi.1005635)
Supplement: S1 File — Simulations were run using a CompuCell3D installation available from the Indiana University Bloomington and the Biocomplexity Institute (www.compucell3d.org) [39]. Parameters for the simulations are set in XML scripts (simulation.xml). Simulations require initial configuration files for cells and diffusible concentration fields (PIFtemplate.pif, FieldGLUtemplate.txt, FieldOXYtemplate.txt) specified in the XML file, and a set of customized plugins (called ‘steppables’ and ‘plugins’) to be installed in the CompuCell3D ‘DeveloperZone’. Simulation outputs are a set of files for each sampling time point that contain cell-based information (values of mutating parameters and other cell-level indicators), concentration field data, and cell configuration data. (ZIP) [file pcbi.1005635.s004.zip › code/SzaboMerksPLoSCompBiol-sim_parameters_explained.pdf]

Supplementary Information to: Blood Vessel Tortuosity Selects against Evolution of Agressive Tumor Cells in Confined Tissue Environments: a Modeling Approach

András Szabó[1*¤] and Roeland M. H. Merks[1,2]

1 Life Sciences Group, Centrum Wiskunde & Informatica, Amsterdam, The Netherlands **2** Mathematical Institute, Leiden University, Leiden, The Netherlands

¤Current Address: Department of Cell and Developmental Biology, UCL, London, United Kingdom
* A.Szabo@ucl.ac.uk

Explanation of simulation parameters

Parameters for simulation runs can be set through an xml script file for CompuCell3D. The script requires that all the custom plugins and steppables are installed in the DeveloperZone of CompuCell3D. This documents shows an example xml script explaining the connection of the simulation parameters and the parameters presented in the corresponding article.

```
<CompuCell3D>
    <Potts>                                                   General cellular Potts model parameters
        <Dimensions x=”200” y=”200” z=”1”/>                   Lattice dimensions
        <Anneal>0</Anneal>
        <Steps>100001</Steps>                                 Simulation time in MCS
        <FluctuationAmplitude>1</FluctuationAmplitude>
        <NeighborOrder>2</NeighborOrder>
        <Boundary_x>Periodic</Boundary_x>
        <Boundary_y>Periodic</Boundary_y>
        <RandomSeed>14273</RandomSeed>                        Change seed for independent simulation runs
        <DebugOutputFrequency>100</DebugOutputFrequency>
    </Potts>
    <Plugin Name=”CellType”>                                  Defining cell types
        <CellType TypeName=”Medium” TypeId=”0”/>
        <CellType TypeName=”Cell” TypeId=”1”/>
        <CellType TypeName=”Endothelial” TypeId=”2” Freeze=””/>
        <CellType TypeName=”BlockedEndothelial” TypeId=”3” Freeze=””/>
    </Plugin>
List of plugins
    <Plugin Name=”CenterOfMass”/>
    <Plugin Name=”BoundaryPixelTracker”>
        <NeighborOrder>2</NeighborOrder>
    </Plugin>
    <Plugin Name=”NeighborTracker”/>
    <Plugin Name=”VolumeImproved”>
        <relativeVolume/>
    </Plugin>
    <Plugin Name=”Contact”>                                   Adhesions due to surface tension (Eq. 7)
```

```
    <Energy Type1="Medium" Type2="Medium">0</Energy>
    <Energy Type1="Medium" Type2="Cell">50</Energy>
    <Energy Type1="Medium" Type2="Endothelial">50</Energy>
    <Energy Type1="Medium" Type2="BlockedEndothelial">50</Energy>
    <Energy Type1="Cell" Type2="Cell">100</Energy>
    <Energy Type1="Cell" Type2="Endothelial">100</Energy>
    <Energy Type1="Cell" Type2="BlockedEndothelial">100</Energy>
    <Energy Type1="Endothelial" Type2="Endothelial">100</Energy>
    <Energy Type1="Endothelial" Type2="BlockedEndothelial">100</Energy>
    <Energy Type1="BlockedEndothelial" Type2="BlockedEndothelial">100</Energy>
    <NeighborOrder>2</NeighborOrder>
</Plugin>

<Plugin Name="AdhesionFlexCopy" >                                   Adhesion due to adhesion
    molecules (Eq. 7). Note: the actual density values are set in
    EvolutionaryExtensionA steppable below
    <AdhesionMolecule Molecule="CAM" />
    <AdhesionMolecule Molecule="MAM" />
    <AdhesionMolecule Molecule="MAS" />
    <AdhesionMoleculeDensity CellType="Medium" Molecule="CAM" Density="0.0"/>
    <AdhesionMoleculeDensity CellType="Medium" Molecule="MAM" Density="0.0"/>
    <AdhesionMoleculeDensity CellType="Medium" Molecule="MAS" Density="50.0"/>
    <AdhesionMoleculeDensity CellType="Cell" Molecule="CAM" Density="99"/>
    <AdhesionMoleculeDensity CellType="Cell" Molecule="MAM" Density="49.5"/>
    <AdhesionMoleculeDensity CellType="Cell" Molecule="MAS" Density="0.0"/>
    <AdhesionMoleculeDensity CellType="Endothelial" Molecule="CAM" Density="
        100.0"/>
    <AdhesionMoleculeDensity CellType="Endothelial" Molecule="MAM" Density="50.0
        "/>
    <AdhesionMoleculeDensity CellType="Endothelial" Molecule="MAS" Density="0.0"
        />
    <AdhesionMoleculeDensity CellType="BlockedEndothelial" Molecule="CAM"
        Density="100.0"/>
    <AdhesionMoleculeDensity CellType="BlockedEndothelial" Molecule="MAM"
        Density="50.0"/>
    <AdhesionMoleculeDensity CellType="BlockedEndothelial" Molecule="MAS"
        Density="0.0"/>
    <BindingFormula Name="Binary">                                  Defining k_{a,b} (Eq. 7)
        <Formula> min(Molecule1,Molecule2)</Formula>
        <Variables>
            <AdhesionInteractionMatrix>
                <BindingParameter Molecule1="CAM" Molecule2="CAM">1.0</
                    BindingParameter>
                <BindingParameter Molecule1="CAM" Molecule2="MAM">0.0</
                    BindingParameter>
                <BindingParameter Molecule1="CAM" Molecule2="MAS">0.0</
                    BindingParameter>
                <BindingParameter Molecule1="MAM" Molecule2="MAM">0.0</
                    BindingParameter>
                <BindingParameter Molecule1="MAM" Molecule2="MAS">1.0</
                    BindingParameter>
```

```
                <BindingParameter Molecule1="MAS" Molecule2="MAS">0.0</
                    BindingParameter>
            </AdhesionInteractionMatrix>
        </Variables>
    </BindingFormula>
    <NeighborOrder>2</NeighborOrder>
</Plugin>
```

Note: actual values are set below

```
<Plugin Name="ChemotaxisCopy">
    <ChemicalField Source="FlexibleDiffusionSolverFE" Name="FGF">
        <ChemotaxisByType Type="Cell" Lambda="0.0"/>
    </ChemicalField>
    <ChemicalField Source="KernelDiffusionSolver" Name="Oxygen">
        <ChemotaxisByType Type="Cell" Lambda="0.0"/>
    </ChemicalField>
    <ChemicalField Source="KernelDiffusionSolver" Name="Glucose">
        <ChemotaxisByType Type="Cell" Lambda="0.0"/>
    </ChemicalField>
    <ChemicalField Source="KernelDiffusionSolver" Name="Lactate">
        <ChemotaxisByType Type="Cell" Lambda="0.0"/>
    </ChemicalField>
</Plugin>
```

List of steppables

Input-output steppables, use absolute paths

Init. cell config

```
<Steppable Type="PIFInitializer">
        <PIFName>PIFtemplate.pif</PIFName>
</Steppable>
```

Conc. fields

```
<Steppable Type="SaveField" Frequency="200">
        <NumberOfFields>3</NumberOfFields>
        <BaseName>output/oxygen,output/glucose,output/lactate</BaseName>
        <FieldName>Oxygen,Glucose,Lactate</FieldName>
</Steppable>
```

Cellular data

```
<Steppable Type="SaveData" Frequency="200">
        <Basename>output/CD_</Basename>
        <ValidForCellTypes>1,2</ValidForCellTypes>
        <Data>all</Data>
        <phylogeny>output/phyl_</phylogeny>
</Steppable>
```

Cell configs

```
<Steppable Type="PIFDumper" Frequency="200">
            <PIFName>output/cells_</PIFName>
</Steppable>
```

Other steppables

```
<Steppable Type="Mitosis" Frequency="1"/>
<Steppable Type="CellManagement" Frequency="1">
    <ValidForCellTypes>1</ValidForCellTypes>
```

For cells only

```
    <MCSinSeconds>1</MCSinSeconds>
```

This is only used for scaling the growth conversion factor, keep value=1.

Initial regime settings (see Fig. 1)

```
    <wait_for_static_fields>
        <GlucoseThreshold>0.05</GlucoseThreshold>
```

```
            <OxygenThreshold>0.05</OxygenThreshold>
            <TimeDifference>100</TimeDifference>
        </wait_for_static_fields>
        <InitialMutation/>
        <DNARepairProb value="1.0">
            <Pmut>0</Pmut>
```

Mutation probability μ

```
            <mode>random</mode>
            <scale>linear</scale>
            <stepsize>0.05</stepsize>
```

Mutation step size σ_p

```
            <min>0</min>
```

Limits of parameter values

```
            <max>1</max>
```

Note: relfective boundaries (Fig. 1)

```
        </DNARepairProb>
```

Blood vessel blocking and unblocking probabilities, $\mathcal{P}$:

```
        <blockEndothelialProbability>0.5</blockEndothelialProbability>
        <releaseEndothelialProbability>0.5</releaseEndothelialProbability>
```

Constantly decreasing switch probability (inactive if zero):

```
        <ECswitchStart>0</ECswitchStart>
        <ECswitchEnd>0</ECswitchEnd>
        <ECswitchMultiplier>0</ECswitchMultiplier>
```

$$\text{multiplier} = \left(\frac{\text{end}}{\text{start}}\right)^{1/\text{time}}$$

For example: $0.5 \to 0.001$ in 100,000 MCS: multiplier = 0.999938

Switch probability depending on tissue coverage (inactive if both zero):

```
        <ECswitchMaxCoverage>0.0</ECswitchMaxCoverage>
        <ECswitchMinCoverage>0.0</ECswitchMinCoverage>
```

Periodic switching (inactive if zero):

```
        <periodOfEndothelialFunction>0</periodOfEndothelialFunction>
```

Initial randomization of EC (probability of converting cell to EC at start):

```
        <randomEndothelialAssignmentActive>0</randomEndothelialAssignmentActive>
        <randomEndothelialAssignmentPassive>0</randomEndothelialAssignmentPassive>

        <showfields Frequency="1000">
```

Display

```
            <showCellClones/>
            <showMinMedContact/>
            <showGrowth/>
            <showNMut/>
            <showGeneration/>
            <showTargetVolume/>
            <showPressure/>
            <showPressureSens/>
            <showROSsens/>
            <showEnergyToUse/>
        </showfields>
        <printCloneDistribution>1000</printCloneDistribution>
```

Print phylogeny/inheritance information

```
    </Steppable>
```

```
<Steppable Type="Metabolism" Frequency="1">
    <ValidForCellTypes>1</ValidForCellTypes>
    <GLUT_Vmax>1e-4</GLUT_Vmax>                                   $V^m(i,t)$ value (Eq. 10)
    <GLUT_Km>2e-4</GLUT_Km>                                       $K(i,t)$ value (Eq. 10)
    <uptakeExtraTimes>50</uptakeExtraTimes>                       Note:
                  uptakeExtraTimes+1 should be greater than $(20 \times V^m N_0(\text{max})/K)$,
                  otherwise the uptake might result in overshoot. The amount of overshoot
                  is displayed in the gluerror and oxyerror fields. If there is still an
                  overshoot, the cell takes up all available substances.

    <fermentorAdvantage>0</fermentorAdvantage>
    <MaintenanceRate>0.5</MaintenanceRate>                        $E_m$ (Eq. 12)
    <GLUTExpression value="250">                                  $N_0$ (Eq. 13)
        <Pmut>0.2</Pmut>
        <mode>diffusive</mode>
        <scale>linear</scale>
        <stepsize>25</stepsize>
        <min>0</min>
        <max>2500</max>                                           $N_0(\text{max})$
    </GLUTExpression>
    <HIF_oxyThreshold value="0.1">                                $\kappa_h$ (Eq. 15)
        <Pmut>0.2</Pmut>
        <mode>diffusive</mode>
        <scale>linear</scale>
        <stepsize>0.001</stepsize>
        <min>0.0001</min>
        <max>10</max>
    </HIF_oxyThreshold>
    <HIF_oxySensitivity value="10">                               $n_h$ (Eq. 15)
        <Pmut>0.0</Pmut>
        <mode>diffusive</mode>
        <scale>linear</scale>
        <stepsize>0.1</stepsize>
        <min>1</min>
        <max>10</max>
    </HIF_oxySensitivity>
    <HIF_rosThreshold value="0.5">                                $\kappa_\zeta$ (Eq. 15)
        <Pmut>0.2</Pmut>
        <mode>diffusive</mode>
        <scale>linear</scale>
        <stepsize>0.01</stepsize>
        <min>0.0001</min>
        <max>100</max>
    </HIF_rosThreshold>
    <HIF_rosSensitivity value="10">                               $n_\zeta$ (Eq. 15)
        <Pmut>0.0</Pmut>
        <mode>diffusive</mode>
        <scale>linear</scale>
```

```
            <stepsize>0.1</stepsize>
            <min>1</min>
            <max>10</max>
        </HIF_rosSensitivity>
        <ROSdecay>0.1</ROSdecay>                                  $\omega_\zeta$ (Eq. 16)
        <maxATPcontent>100</maxATPcontent>                        Limit to cell energy
        <Initial_oxygen>0.0</Initial_oxygen>                      Initial
        <Initial_glucose>0.0</Initial_glucose>                    intracellular
        <Initial_lactate>0</Initial_lactate>                      values
        <Initial_ATP>0</Initial_ATP>
        <print_field_sums>100</print_field_sums>                  Outputs
        <showfields Frequency="1000">
                <showATPfield/>
                <showGlucoseUptake/>
                <showOxygenUptake/>
                <showATPproduction/>
        </showfields>
    </Steppable>

    <Steppable Type="CellGrowthControl" Frequency="1">
        <ValidForCellTypes>1</ValidForCellTypes>
        <ConversionFactor>1</ConversionFactor>                    $\alpha$ (Eq. 18)
        <energyToUse value="1.0">                                 Ratio of energy to use
            <Pmut>0.0</Pmut>
            <mode>diffusive</mode>
            <scale>linear</scale>
            <stepsize>0.001</stepsize>
            <min>0.0</min>
            <max>1</max>
        </energyToUse>
        <PressureCoefficient value="0.01">                        $\beta$ (Eq. 18)
            <Pmut>0.0</Pmut>
            <mode>diffusive</mode>
            <scale>linear</scale>
            <stepsize>0.1</stepsize>
            <min>0</min>
            <max>100</max>
        </PressureCoefficient>
        <ShrinkRate>5e0</ShrinkRate>                              Controlled shrinking
        <ROSsensitivity value="1">                                $\gamma$ (Eq. 18)
            <Pmut>0.0</Pmut>
            <mode>diffusive</mode>
            <scale>linear</scale>
            <stepsize>1</stepsize>
            <min>0</min>
            <max>100</max>
        </ROSsensitivity>
        <ROSthreshold value="0">                                  $\theta_R$ (Eq. 18)
            <Pmut>0.0</Pmut>
```

```
            <mode>diffusive</mode>
            <scale>linear</scale>
            <stepsize>1</stepsize>
            <min>0</min>
            <max>100</max>
        </ROSthreshold>
```

V^T

```
        <TargetVolume>25</TargetVolume>
```

V_D (Eq. 19)

```
        <DoublingVolume value="50">
            <Pmut>0.2</Pmut>
            <mode>diffusive</mode>
            <scale>linear</scale>
            <stepsize>1</stepsize>
            <min>10</min>
            <max>200</max>
        </DoublingVolume>
```

λ_v (Eq. 3)

```
        <LambdaVolume value="1">
            <Pmut>0.2</Pmut>
            <mode>diffusive</mode>
            <scale>linear</scale>
            <stepsize>0.1</stepsize>
            <min>0</min>
            <max>20</max>
        </LambdaVolume>
```

Potential limitation to cell divisions

```
        <MaxDivisions value="-1">
            <Pmut>0</Pmut>
            <mode>diffusive</mode>
            <scale>linear</scale>
            <stepsize>100</stepsize>
            <min>-1</min>
            <max>-1</max>
        </MaxDivisions>
```

V^T initially asynchronous

```
        <InitRandomVolume/>
```

Outputs

```
        <print_statistics>
            <frequency>100</frequency>
            <cellVolumes/>
            <cellTargetVolumes/>
            <cellNumbers/>
        </print_statistics>
    </Steppable>

    <Steppable Type="CellSignaling" Frequency="1">
```

Potential growth limitation

```
        <MinMediumContactForGrowth value="0">
            <Pmut>0.0</Pmut>
            <mode>diffusive</mode>
            <scale>linear</scale>
            <stepsize>1</stepsize>
            <min>0</min>
            <max>20</max>
        </MinMediumContactForGrowth>
```

Basic turnover rate

```
        <RandomCellDeath>1e-3</RandomCellDeath>
```

```
    <RandomCellDeathIncrease>0</RandomCellDeathIncrease>
```

Possible increase in death rate, used when cells are dividing fast

```
    <RandomCellDeathK>2.0</RandomCellDeathK>
```

Rate of growth above which the probability of killing a cell is higher

```
    <minCellSize>5</minCellSize>
</Steppable>

<Steppable Type="EvolutionaryTumorExtensionA" Frequency="1">
```

Module for setting and evolving adhesion molecule densities

```
    <AdhesionMoleculeDensity CellType="Medium" Molecule="CAM" Density="0.0">
        <value>0</value>
        <Pmut>0</Pmut>
        <mode>diffusive</mode>
        <scale>linear</scale>
        <stepsize>0</stepsize>
        <min>0</min>
        <max>0</max>
    </AdhesionMoleculeDensity>
    <AdhesionMoleculeDensity CellType="Medium" Molecule="MAM" Density="0.0">
        <value>0</value>
        <Pmut>0</Pmut>
        <mode>diffusive</mode>
        <scale>linear</scale>
        <stepsize>0</stepsize>
        <min>0</min>
        <max>0</max>
    </AdhesionMoleculeDensity>
    <AdhesionMoleculeDensity CellType="Medium" Molecule="MAS" Density="50.0">
```

$\rho_{\mathrm{MAS}}(i)\,|_{\tau(i)=\mathrm{m}}$ (Eq. 7)

```
        <value>50</value>
        <Pmut>0</Pmut>
        <mode>diffusive</mode>
        <scale>linear</scale>
        <stepsize>0.05</stepsize>
        <min>0</min>
        <max>50</max>
    </AdhesionMoleculeDensity>
    <AdhesionMoleculeDensity CellType="Cell" Molecule="CAM" Density="99">
```

$\rho_{\mathrm{CAM}}(i)\,|_{\tau(i)=\mathrm{c}}$ (Eq. 7)

```
        <value>99</value>
        <Pmut>0.2</Pmut>
        <mode>diffusive</mode>
        <scale>linear</scale>
        <stepsize>0.1</stepsize>
        <min>0</min>
        <max>100</max>
    </AdhesionMoleculeDensity>
    <AdhesionMoleculeDensity CellType="Cell" Molecule="MAM" Density="49.5">
```

$\rho_{\mathrm{MAM}}(i)\,|_{\tau(i)=\mathrm{c}}$ (Eq. 7)

```
        <value>49.5</value>
```

```
        <Pmut>0.2</Pmut>
        <mode>diffusive</mode>
        <scale>linear</scale>
        <stepsize>0.1</stepsize>
        <min>0</min>
        <max>50</max>
    </AdhesionMoleculeDensity>
    <AdhesionMoleculeDensity CellType="Cell" Molecule="MAS" Density="0.0">
        <value>0</value>
        <Pmut>0</Pmut>
        <mode>diffusive</mode>
        <scale>linear</scale>
        <stepsize>0.05</stepsize>
        <min>0</min>
        <max>0</max>
    </AdhesionMoleculeDensity>
    <AdhesionMoleculeDensity CellType="Endothelial" Molecule="CAM" Density="
       100.0">
```

$\rho_{\mathrm{CAM}}(i)\mid_{\tau(i)=\mathrm{EC}}$ (Eq. 7)

```
        <value>100</value>
        <Pmut>0</Pmut>
        <mode>diffusive</mode>
        <scale>linear</scale>
        <stepsize>0</stepsize>
        <min>100</min>
        <max>100</max>
    </AdhesionMoleculeDensity>
    <AdhesionMoleculeDensity CellType="Endothelial" Molecule="MAM" Density="50.0
       ">
```

$\rho_{\mathrm{MAM}}(i)\mid_{\tau(i)=\mathrm{EC}}$ (Eq. 7)

```
        <value>50</value>
        <Pmut>0</Pmut>
        <mode>diffusive</mode>
        <scale>linear</scale>
        <stepsize>0</stepsize>
        <min>50</min>
        <max>50</max>
    </AdhesionMoleculeDensity>
    <AdhesionMoleculeDensity CellType="Endothelial" Molecule="MAS" Density="0.0"
       >
        <value>0</value>
        <Pmut>0</Pmut>
        <mode>diffusive</mode>
        <scale>linear</scale>
        <stepsize>0</stepsize>
        <min>0</min>
        <max>0</max>
    </AdhesionMoleculeDensity>
    <AdhesionMoleculeDensity CellType="BlockedEndothelial" Molecule="CAM"
       Density="100.0">
        <value>100</value>
        <Pmut>0</Pmut>
```

```
            <mode>diffusive</mode>
            <scale>linear</scale>
            <stepsize>0</stepsize>
            <min>100</min>
            <max>100</max>
        </AdhesionMoleculeDensity>
        <AdhesionMoleculeDensity CellType="BlockedEndothelial" Molecule="MAM"
            Density="50.0">
            <value>50</value>
            <Pmut>0</Pmut>
            <mode>diffusive</mode>
            <scale>linear</scale>
            <stepsize>0</stepsize>
            <min>50</min>
            <max>50</max>
        </AdhesionMoleculeDensity>
        <AdhesionMoleculeDensity CellType="BlockedEndothelial" Molecule="MAS"
            Density="0.0">
            <value>0</value>
            <Pmut>0</Pmut>
            <mode>diffusive</mode>
            <scale>linear</scale>
            <stepsize>0</stepsize>
            <min>0</min>
            <max>0</max>
        </AdhesionMoleculeDensity>
    </Steppable>

    <Steppable Type="EvolutionaryTumorExtensionB" Frequency="1">
```

Module for setting and evolving chemotaxis parameters

```
        <ChemicalField Source="FlexibleDiffusionSolverFE" Name="FGF">
            <ChemotaxisByType Type="Cell">
                <value>0</value>
                <Pmut>0</Pmut>
                <mode>diffusive</mode>
                <scale>linear</scale>
                <stepsize>0</stepsize>
                <min>-20</min>
                <max>20</max>
            </ChemotaxisByType>
        </ChemicalField>
        <ChemicalField Source="KernelDiffusionSolver" Name="Oxygen">
            <ChemotaxisByType Type="Cell">
```

χ_{O_2} (Eq. 4)

```
                <value>0</value>
                <Pmut>0.2</Pmut>
                <mode>diffusive</mode>
                <scale>linear</scale>
                <stepsize>0.1</stepsize>
                <min>-20</min>
                <max>20</max>
            </ChemotaxisByType>
```

```
        </ChemicalField>
        <ChemicalField Source="KernelDiffusionSolver" Name="Glucose">
            <ChemotaxisByType Type="Cell">
```

χ_g (Eq. 4)

```
                <value>0</value>
                <Pmut>0.2</Pmut>
                <mode>diffusive</mode>
                <scale>linear</scale>
                <stepsize>0.1</stepsize>
                <min>-20</min>
                <max>20</max>
            </ChemotaxisByType>
        </ChemicalField>
        <ChemicalField Source="KernelDiffusionSolver" Name="Lactate">
            <ChemotaxisByType Type="Cell">
```

χ_l (Eq. 4)

```
                <value>0</value>
                <Pmut>0.2</Pmut>
                <mode>diffusive</mode>
                <scale>linear</scale>
                <stepsize>0.1</stepsize>
                <min>-20</min>
                <max>20</max>
            </ChemotaxisByType>
        </ChemicalField>
    </Steppable>
```

Steppables related to diffusive media

```
    <Steppable Type="KernelDiffusionSolver">
        <DiffusionField>
            <Kernel>10</Kernel>
            <CoarseGrainFactor>2</CoarseGrainFactor>
            <DiffusionData>
                <FieldName>Oxygen</FieldName>
```

Diffusion constant for oxygen: $10^{-9} m^2/s$;

the parameter is in pixel^2/MCS, with 1 pixel=2μm, 1MCS=1min, therefore:

```
                <DiffusionConstant>15000</DiffusionConstant>
                <ConcentrationFileName>FieldOXYtemplate.txt</ConcentrationFileName>
```

Initial concentrations, use absolute path

```
            </DiffusionData>

            <SecretionData>
                <ConstantConcentration Type="Endothelial">10</ConstantConcentration>
```

Constant concentration within active blood vessels

```
                <Secretion Type="Medium">0.0</Secretion>
                <Secretion Type="Cell">0.0</Secretion>
                <Secretion Type="BlockedEndothelial">0.0</Secretion>
            </SecretionData>
        </DiffusionField>
```

```
        <DiffusionField>
            <Kernel>10</Kernel>
            <CoarseGrainFactor>2</CoarseGrainFactor>
            <DiffusionData>
                <FieldName>Glucose</FieldName>
```

Diffusion constant for glucose = $10^{-11} m^2/s$:

```
                <DiffusionConstant>150</DiffusionConstant>
                <DecayConstant>0.0</DecayConstant>
                <ConcentrationFileName>FieldGLUtemplate.txt</ConcentrationFileName>
            </DiffusionData>
            <SecretionData>
                <ConstantConcentration Type="Endothelial">10</ConstantConcentration>
                <Secretion Type="Medium">0.0</Secretion>
                <Secretion Type="Cell">0.0</Secretion>
                <Secretion Type="BlockedEndothelial">0.0</Secretion>
           </SecretionData>
        </DiffusionField>

        <DiffusionField>
            <Kernel>10</Kernel>
            <CoarseGrainFactor>2</CoarseGrainFactor>
            <DiffusionData>
                <FieldName>Lactate</FieldName>
```

Diffusion constant for lactate = $10^{-11} m^2/s$

```
                <DiffusionConstant>150</DiffusionConstant>
                <DecayConstant>0.0</DecayConstant>
            </DiffusionData>
            <SecretionData>
                <ConstantConcentration Type="Endothelial">0</ConstantConcentration>
            </SecretionData>
        </DiffusionField>
    </Steppable>
```

Proxy concentration fields, used for display and technicalities

```
    <Steppable Type="FlexibleDiffusionSolverFE">
        <DiffusionField>
            <DiffusionData>
                <FieldName>FGF</FieldName>
                <DiffusionConstant>0.0</DiffusionConstant>
                <DecayConstant>0.0</DecayConstant>
            </DiffusionData>
        </DiffusionField>
        <DiffusionField>
            <DiffusionData>
                <FieldName>ATP</FieldName>
                <DiffusionConstant>0.0</DiffusionConstant>
                <DecayConstant>0.0</DecayConstant>
            </DiffusionData>
        </DiffusionField>
        <DiffusionField>
            <DiffusionData>
                <FieldName>GlucoseUptake</FieldName>
```

```
            <DiffusionConstant>0.0</DiffusionConstant>
            <DecayConstant>0.0</DecayConstant>
        </DiffusionData>
    </DiffusionField>
    <DiffusionField>
        <DiffusionData>
            <FieldName>OxygenUptake</FieldName>
            <DiffusionConstant>0.0</DiffusionConstant>
            <DecayConstant>0.0</DecayConstant>
        </DiffusionData>
    </DiffusionField>
    <DiffusionField>
        <DiffusionData>
            <FieldName>ATPproduction</FieldName>
            <DiffusionConstant>0.0</DiffusionConstant>
            <DecayConstant>0.0</DecayConstant>
        </DiffusionData>
    </DiffusionField>
    <DiffusionField>
        <DiffusionData>
            <FieldName>CellClones</FieldName>
            <DiffusionConstant>0.0</DiffusionConstant>
            <DecayConstant>0.0</DecayConstant>
        </DiffusionData>
    </DiffusionField>
    <DiffusionField>
        <DiffusionData>
            <FieldName>Growth</FieldName>
            <DiffusionConstant>0.0</DiffusionConstant>
            <DecayConstant>0.0</DecayConstant>
        </DiffusionData>
    </DiffusionField>
    <DiffusionField>
        <DiffusionData>
            <FieldName>TargetVolume</FieldName>
            <DiffusionConstant>0.0</DiffusionConstant>
            <DecayConstant>0.0</DecayConstant>
        </DiffusionData>
    </DiffusionField>
    <DiffusionField>
        <DiffusionData>
            <FieldName>Pressure</FieldName>
            <DiffusionConstant>0.0</DiffusionConstant>
            <DecayConstant>0.0</DecayConstant>
        </DiffusionData>
    </DiffusionField>
    <DiffusionField>
        <DiffusionData>
            <FieldName>HIF</FieldName>
            <DiffusionConstant>0.0</DiffusionConstant>
            <DecayConstant>0.0</DecayConstant>
        </DiffusionData>
```

```
    </DiffusionField>
    <DiffusionField>
        <DiffusionData>
            <FieldName>ROS</FieldName>
            <DiffusionConstant>0.0</DiffusionConstant>
            <DecayConstant>0.0</DecayConstant>
        </DiffusionData>
    </DiffusionField>
    <DiffusionField>
        <DiffusionData>
            <FieldName>GLUT</FieldName>
            <DiffusionConstant>0.0</DiffusionConstant>
            <DecayConstant>0.0</DecayConstant>
        </DiffusionData>
    </DiffusionField>
    <DiffusionField>
        <DiffusionData>
            <FieldName>NMut</FieldName>
            <DiffusionConstant>0.0</DiffusionConstant>
            <DecayConstant>0.0</DecayConstant>
        </DiffusionData>
    </DiffusionField>
    <DiffusionField>
        <DiffusionData>
            <FieldName>PressureSens</FieldName>
            <DiffusionConstant>0.0</DiffusionConstant>
            <DecayConstant>0.0</DecayConstant>
        </DiffusionData>
    </DiffusionField>
    <DiffusionField>
        <DiffusionData>
            <FieldName>ROSsens</FieldName>
            <DiffusionConstant>0.0</DiffusionConstant>
            <DecayConstant>0.0</DecayConstant>
        </DiffusionData>
    </DiffusionField>
    <DiffusionField>
        <DiffusionData>
            <FieldName>Generation</FieldName>
            <DiffusionConstant>0.0</DiffusionConstant>
            <DecayConstant>0.0</DecayConstant>
        </DiffusionData>
    </DiffusionField>
    <DiffusionField>
        <DiffusionData>
            <FieldName>oxyerror</FieldName>
            <DiffusionConstant>0.0</DiffusionConstant>
            <DecayConstant>0.0</DecayConstant>
        </DiffusionData>
    </DiffusionField>
    <DiffusionField>
        <DiffusionData>
```

```
                <FieldName>gluerror</FieldName>
                <DiffusionConstant>0.0</DiffusionConstant>
                <DecayConstant>0.0</DecayConstant>
            </DiffusionData>
        </DiffusionField>
        <DiffusionField>
            <DiffusionData>
                <FieldName>MinMedContact</FieldName>
                <DiffusionConstant>0.0</DiffusionConstant>
                <DecayConstant>0.0</DecayConstant>
            </DiffusionData>
        </DiffusionField>
        <DiffusionField>
            <DiffusionData>
                <FieldName>ActualGLUT</FieldName>
                <DiffusionConstant>0.0</DiffusionConstant>
                <DecayConstant>0.0</DecayConstant>
            </DiffusionData>
        </DiffusionField>
        <DiffusionField>
            <DiffusionData>
                <FieldName>EnergyToUse</FieldName>
                <DiffusionConstant>0.0</DiffusionConstant>
                <DecayConstant>0.0</DecayConstant>
            </DiffusionData>
        </DiffusionField>
    </Steppable>


</CompuCell3D>
```
